# Superior breast cancer metastasis risk stratification using an epithelial-mesenchymal-amoeboid transition gene signature

**DOI:** 10.1186/s13058-020-01304-8

**Published:** 2020-07-08

**Authors:** Amin Emad, Tania Ray, Tor W. Jensen, Meera Parat, Rachael Natrajan, Saurabh Sinha, Partha S. Ray

**Affiliations:** 1 Department of Electrical and Computer Engineering, McGill University, Montreal, Quebec Canada; 2 Carl R. Woese Institute for Genomic Biology, University of Illinois at Urbana-Champaign, Champaign, Illinois USA; 3 Department of Computer Science, University of Illinois at Urbana-Champaign, Champaign, Illinois USA; 4 Onconostic Technologies Inc., Champaign, Illinois USA; 5 Illinois Health Sciences Institute, University of Illinois at Urbana-Champaign, Champaign, Illinois USA; 6 The Breast Cancer Now Toby Robins Research Centre, The Institute of Cancer Research, London, UK; 7 Cancer Center at Illinois, University of Illinois at Urbana-Champaign, Champaign, Illinois USA

**Keywords:** Epithelial-to-mesenchymal transition, Mesenchymal-to-amoeboid transition, Metastasis, Breast cancer subtypes, Metastatic risk assessment

## Abstract

**Background:**

Cancer cells are known to display varying degrees of metastatic propensity, but the molecular basis underlying such heterogeneity remains unclear. Our aims in this study were to (i) elucidate prognostic subtypes in primary tumors based on an epithelial-to-mesenchymal-to-amoeboid transition (EMAT) continuum that captures the heterogeneity of metastatic propensity and (ii) to more comprehensively define biologically informed subtypes predictive of breast cancer metastasis and survival in lymph node-negative (LNN) patients.

**Methods:**

We constructed a novel metastasis biology-based gene signature (EMAT) derived exclusively from cancer cells induced to undergo either epithelial-to-mesenchymal transition (EMT) or mesenchymal-to-amoeboid transition (MAT) to gauge their metastatic potential. Genome-wide gene expression data obtained from 913 primary tumors of lymph node-negative breast cancer (LNNBC) patients were analyzed. EMAT gene signature-based prognostic stratification of patients was performed to identify biologically relevant subtypes associated with distinct metastatic propensity.

**Results:**

Delineated EMAT subtypes display a biologic range from less stem-like to more stem-like cell states and from less invasive to more invasive modes of cancer progression. Consideration of EMAT subtypes in combination with standard clinical parameters significantly improved survival prediction. EMAT subtypes outperformed prognosis accuracy of receptor or PAM50-based BC intrinsic subtypes even after adjusting for treatment variables in 3 independent, LNNBC cohorts including a treatment-naïve patient cohort.

**Conclusions:**

EMAT classification is a biologically informed method that provides prognostic information beyond that which can be provided by traditional cancer staging or PAM50 molecular subtype status and may improve metastasis risk assessment in early stage, LNNBC patients, who may otherwise be perceived to be at low metastasis risk.

## Introduction

Metastasis is one of the key hallmarks of cancer [1] and accounts for nearly 90% of cancer-related mortality. Cancer cells within a tumor are known to possess different metastatic potentials [2]. However, the molecular basis underlying the observed heterogeneity in metastatic proclivity remains unclear and a suitable molecular classification is lacking. Intrinsic molecular subtypes of breast cancer (PAM50), that have provided tremendous insight regarding the biological origins of breast cancer, have been associated with distinct metastatic predilections for one organ or the other [3], but the PAM50 classification is independent of susceptibility to metastatic spread [4]. For instance, an intrinsic subtype that displays a higher rate of brain metastasis does not necessarily mean all patients, or even the majority of patients diagnosed with that subtype of cancer will go on to manifest with metastatic disease in the brain. Clearly other factors, independent of and in addition to those that determine the intrinsic molecular subtype of the cancer, influence its invasive potential and metastatic propensity.

Although implicated in cancer progression and metastasis, the clinical significance of processes like epithelial-to-mesenchymal transition (EMT) and mesenchymal-to-amoeboid transition (MAT) remains to be fully appreciated. EMT, a cellular transformation process that plays a key role in embryonic development, is widely considered to be one such factor influencing metastasis. Cancer cells derepress the normally silenced EMT molecular program, acquiring malignant properties that enable them to invade tissues surrounding their site of origin thereby effectively spreading and colonizing distant sites [5, 6]. Likewise, MAT is another process that plays an important role in embryonic development and is similarly reawakened by cancers during the metastatic cascade [7, 8].

Since the EMT process is exploited by cancer cells progressing to metastasis, there have been several attempts to subtype patient tumors based on an EMT signature, but these have not been successful in demonstrating a discernible difference in associated breast cancer prognosis [9–11]. Additionally, for cancer cells that have already transitioned through EMT but are facing microenvironmental (e.g., hypoxia) or xenobiotic (e.g., chemotherapy) stress, MAT may be an effective adaptive response to bypass the stress [12]. Indeed a recent report of effectively thwarting metastatic spread through simultaneous targeting of both mesenchymal and amoeboid motility in an animal model of cancer progression appears to support this notion [13]. We thus hypothesized that the true clinical and prognostic significance of EMT as a driving process in cancer progression towards distant metastasis cannot be fully appreciated unless it is considered in the context of being complemented by the conditional occurrence of MAT as well. Only when both processes are considered to coexist, and possibly undergo plastic interchange triggered by environmental and/or xenobiotic pressures, can the clinical significance of both be recognized and prognostic impact demonstrated. We therefore sought to develop a more inclusive gene expression signature that accurately captures EMT, MAT, and the variable dynamic co-occurrence of both the processes in the same tumor. In this study, our aims were to (i) elucidate prognostic subtypes in primary tumors based on an EMT-MAT continuum that captures the heterogeneity of metastatic propensity and (ii) to more comprehensively define biologically informed subtypes predictive of breast cancer metastasis and survival in lymph node-negative (LNN) patients.

## Methods

### Derivation of MAT and EMAT gene lists

We constructed an “EMAT” gene signature comprising a list of 253 previously reported EMT-related genes [9] and 138 newly derived MAT-related genes (Supplementary Table S1). The EMT list had originally been derived by analyzing gene expression data obtained from 5 distinct and separate EMT-inducing cell perturbation experiments to identify genes up- or downregulated at least 2-fold in at least 3 experimental groups relative to control cells. Following identical methodology to minimize derivation bias, we derived a new MAT signature by analyzing gene expression data obtained from 4 distinct and separate MAT-inducing cell perturbation experiments [14] to identify 138 genes up- or downregulated at least 1.5-fold in at least 2 experimental groups relative to control cells. We then combined both the EMT and MAT signatures above to create the 388-member EMAT signature.

### Data collection

Gene expression and clinical data for 562 LNN breast cancer patient samples from the METABRIC study was utilized as a training dataset (OASIS http://oasis-genomics.org/, [15]) and two independent datasets of 200 LNN samples and 151 LNN samples were used as test cohorts for validation (http://www.ncbi.nlm.nih.gov/geo/ GEO accession number GSE11121, http://ccb.nki.nl/data/ [16]), as summarized in Table 1. Gene expression profiles of H1 hESC lines [17] were also obtained (GEO accession number GSE54186). In all datasets, the probe intensities were log2 transformed and Z normalized prior to analysis.

**Table 1:** Clinical and pathological characteristics of patient cohorts used to assess prognostic significance of EMAT subtypes

Factor	METABRIC (***n*** = 562) No. (%)	GSE11121 (***n*** = 200) No. (%)	NKI295 (***n*** = 151) No. (%)
Age, years			
< 50	20.8	23.2	84.1
> 50	78.8	76.8	15.9
Unknown	0.4	100	
Tumor size, cm			
< 2	39.5	49.5	54.3
2–5	57.7	49	45.7
> 5	2.3	1.5	
Unknown	0.5	0	0
Tumor grade			
Low	8.5	14.5	22.5
Intermediate	42.3	68	30.5
High	42.3	17.5	47.0
Unknown	6.8	0	0
ER			
Negative	17.3	23.2	27.8
Positive	78.8	76.8	72.2
Unknown	3.9	0	0
PR			
Negative	43.8	41.8	
Positive	56.2	58.2	
Unknown	0	0	100
HER2			
Negative	80.2	89.0	
Positive	19.4	0.11	
Unknown	0.4	0	100
Adjuvant therapy			
Chemotherapy	7.1	N/A	4.0
Hormonal therapy	47.9	N/A	4.0
Radiation therapy	54.4	N/A	40.4
PAM50 status			
Luminal A	44.1	27	31.8
Luminal B	19.9	23.5	25.2
HER2	7.5	12.5	16.6
Basal-like	15.3	17.5	18.5
Normal-like	13	18	7.9
EMAT status			
EMAT1	19.4	22.5	17.2
EMAT2	45.7	31	35.1
EMAT3	26	29.5	27.8
EMAT4	8.9	17	19.9

*ER* estrogen receptor, *HER2* human epidermal growth factor receptor 2, *PR* progesterone receptor

### Hierarchical clustering

Hierarchical clustering of samples was performed using the python module SciPy, using Ward’s variance minimization algorithm. To obtain EMAT clusters, we partitioned METABRIC LNN samples into *n* = 3, 4, and 5 clusters and found that *n* = 4 yields the best grouping of samples based on average silhouette scores [18], when considering three different distance measures (cosine, Euclidean, and correlation).

### Survival analysis

Survival analysis (Kaplan-Meier and Cox regression) was performed using KnowEnG analytical platform (www.knoweng.org) [19], lifelines python module (doi: 10.5281/zenodo.815943), and Survival R package (https://CRAN.R-project.org/package=survival). Variables included in the multivariate analysis were age, tumor size, tumor grade, subtype status based on receptor profile, PAM50 centroid or EMAT centroid, adjuvant chemotherapy, hormone therapy or radiotherapy. All tests were two-sided, and *p* < 0.05 was considered statistically significant.

### Cross-validation framework to evaluate survival predictive ability of different predictors

To compare the predictive ability of different classes of features, we used a cross-validation framework, in which half of the samples (patients) were randomly selected as the training set and the other half were used as a test set. The training set was grouped into *n* = 4 clusters using the EMAT signature. A Cox regression model was trained on these samples, using clinical variables as well as EMAT cluster status. In parallel, Cox regression models were trained on the same samples using the other types of predictors. Each trained model was then used to estimate the expected survival of the remaining samples (i.e., test samples). In this step, EMAT cluster status was assigned to test samples using a centroid-based classifier trained on the training samples. Then, the estimated survival times were compared to the observed survival times using the concordance index (C-index). To ensure the specific choice of training and test set does not influence the results, this process was repeated 200 times, each time using a distinct random partition of data. To determine the EMAT cluster assignment of the test samples, we first used hierarchical clustering to cluster samples in the training set into four clusters. Then we trained a centroid-based classifier on the training set and predicted the cluster labels for the test set. The *p*-values were calculated using a one-sided Wilcoxon signed rank test and represent the significance of the improvement obtained using EMAT cluster status and clinical parameters as compared to other predictors. The measure PIF shows the percent of times in which EMAT + clinical parameters provided a more accurate prediction compared to the baseline.

### The KNN and centroid-based classifiers

We implemented a KNN (only used to generate Supplementary Fig. S7) and a centroid-based classifier for our analyses trained on LNN METABRIC samples. To reduce cross-dataset analysis batch effects and relax the normality assumption for gene expression values, we used the Spearman’s rank correlation as the measure of the sample similarity in these classifiers. In the centroid-based classifier, for each training cluster, a centroid was calculated as a vector in which each entry corresponds to the mean expression of a gene across the samples of the cluster. The cluster (EMAT subtype) whose centroid had the highest Spearman’s rank correlation with the expression profile of a test sample was selected as the sample’s label (Supplementary Table S2).

## Results

### Prognostic significance of EMAT gene signature in breast cancer

**Fig. 1:**
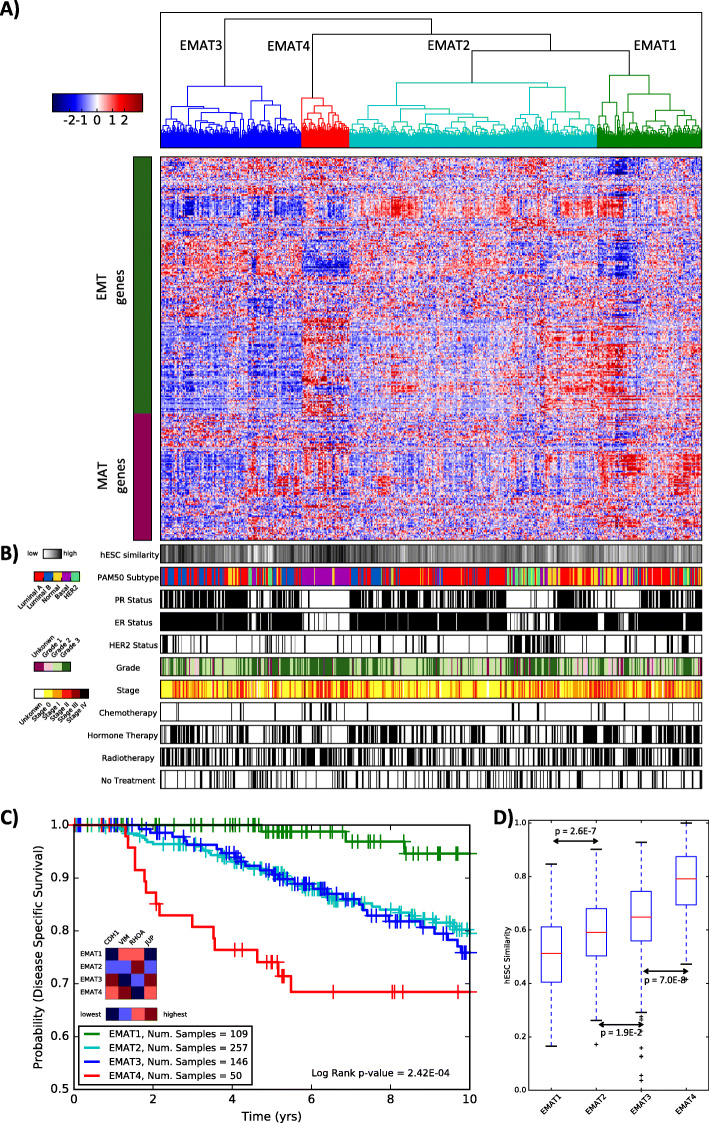
EMAT clusters and their characteristics. **a** EMAT clusters based on lymph node-negative METABRIC samples obtained using hierarchical clustering. The heatmap shows the normalized expression of EMAT genes (rows) in each sample (columns). Sample dendrogram colors are chosen to match those of Kaplan-Meier plots in **c**. **b** Characterization of samples based on similarity to hESC, PAM50 subtypes, ER, PR, and HER2 status, stage, grade, and type of treatment. Spearman’s rank correlation, scaled between 0 and 1 using min-max normalization, is used as the measure of similarity of samples to hESC, in which 0 and 1 represent least similar and most similar, respectively. **c** Kaplan-Meier plots corresponding to *n* = 4 clusters. The heatmap shows the relative ranking of the average expression of four biomarkers in each cluster compared to other clusters. Clusters EMAT2 and EMAT3 have very similar Kaplan-Meier survival curves (**c**), even though their gene expression profiles are distinct. EMAT4 has a worse survival outcome compared to other clusters (*p* = 0.05 against EMAT3, *p* = 0.01 against EMAT2 and *p* = 1.8E-6 against EMAT1). **d** The box plots show the distribution of hESC similarity of the samples in each cluster. The similarity is defined as the Spearman’s rank correlation (scaled between 0 and 1) between expression profiles of H1 hESC lines and each sample. The *p*-values (calculated using a one-sided *t*-test) show how significant the differences between two adjacent EMAT clusters are with respect to their similarity to hESC. The significance *p*-value for the cluster with the least similarity to hESC (EMAT1) and the cluster with the most similarity to hESC (EMAT4) is *p* = 1.7E−23

Hierarchical clustering of patient samples from the METABRIC study using the EMAT gene signature yielded four separate clusters (Fig. 1) with statistically significant separation of associated Kaplan-Meier survival curves (*p* = 2.42E−4, log rank test, Fig. 1c) despite the clustering procedure not being privy to survival data and demonstrated statistically significant prognostic value on both univariable and multivariable Cox regression analysis (Table 2). EMAT clusters displayed distinct expression profiles for CDH1, VIM, RHOA, and JUP, well-accepted biomarkers of epithelial (E), mesenchymal (M), amoeboid (A), and collective cell migration in breast cancer [20], respectively. EMAT4, the cluster with the lowest disease-specific survival (DSS) probability, showed M-like characteristics (VIM was over-expressed in this cluster compared to the other three clusters, *p* = 4.8E−8, unpaired two-tailed *t*-test). Cluster EMAT3 had the lowest average expression of VIM (*p* = 3.7E−79) and the highest average expression of CDH1 (*p* = 1.3E−3) and JUP (*p* = 8.3E−6), suggesting that early onset of epithelial collective cell migration may be manifested in this group of patients. EMAT2 had the highest average expression of RHOA (*p* = 3.8E−3), suggesting A-like characteristics. Finally, EMAT1 had high expressions of VIM and RHOA but low expression of CDH1 and JUP, with the average expression of JUP being smallest (*p* = 3.9E−15) in this cluster. These results indicate existence of clusters having hybrid characteristics rather than discrete E-, M- and A- subtypes and emphasize the advantage of using the EMAT signature over using only E, M, or A biomarkers to distinguish groups of patient tumors associated with distinct prognosis.

**Table 2:** Univariable and multivariable analysis of 562 LNN breast cancer patients (METABRIC dataset [13]) used to examine prognostic value of EMAT subtype designation status for 10-year follow-up

	Univariable analysis			Multivariable (age, tumor size, grade, IHC status**, any chemo/hormonal/ radiation therapy)			Multivariable (age, tumor size, grade, PAM50 status^#^, any chemo/hormonal/ radiation therapy)			Multivariable (age, tumor size, grade, EMAT status^##^, any chemo/hormonal/ radiation therapy)		
	*n*	*P*	HR (95% CI)	*n*	*P*	HR (95% CI)	*n*	*P*	HR (95% CI)	*n*	*P*	HR (95% CI)
Age	562	0.70	1.00 (0.99–1.02)	510	0.18	1.014 (0.99–1.03)	521	0.21	1.01 (0.99–1.03)	521	0.21	1.01 (0.99–1.03)
Tumor size (< 2, 2–5, > 5)	559	0.02	1.67 (1.10–2.54)	510	0.04	1.59 (1.04–2.45)	521	0.03	1.60 (1.04–2.49)	521	0.04	1.59 (1.03–2.47)
Tumor grade*	524	0.20	1.26 (0.89–1.79)	510	0.46	1.16 (0.78–1.71)	521	0.45	1.16 (0.78–1.73)	521	0.98	1.01 (0.69–1.47)
Any chemotherapy	562	0.15	1.78 (0.82–3.87)	510	0.17	1.86 (0.78–4.451)	521	0.12	1.99 (0.84–4.77)	521	0.10	2.07 (0.87–4.92)
Any hormonal therapy	562	0.15	0.72 (0.46–1.12)	510	0.06	0.62 (0.38–1.01)	521	0.04	0.60 (0.37–0.97)	521	0.05	0.62 (0.39–1.00)
Any radiation therapy	562	0.20	0.76 (0.50–1.16)	510	0.28	0.78 (0.50–1.22)	521	0.24	0.77 (0.49–1.19)	521	0.15	0.72 (0.46–1.12)
IHC status**	538	0.03	1.23 (1.02–1.47)	510	0.32	1.11 (0.90–1.37)						
PAM50 status^#^	562	0.12	1.14 (0.97–1.35)				521	0.67	1.04 (0.86–1.27)			
EMAT status^##^	562	1E−4	1.65 (1.29–2.11)							521	3E−4	1.64 (1.25–2.14)

Two-sided *p*-values were based on *χ*^2^ or Fisher’s exact test, whenever appropriate

*CI* confidence interval, *ER* estrogen receptor, *HER2* human epidermal growth factor receptor 2, *HR* hazard ratio, *PR* progesterone receptor, *P p*-value, *n* number of samples

*(Low - “1,” intermediate - “2,” high - “3”)

**(ER+HER2− “1,” ER+HER2+ “2,” ER−HER2+ “3,” ER−HER2− “4”)

^#^(Normal-like - “0,” LumA - “1,” LumB - “2,” HER2 - “3,” basal-like - “4”)

^##^(EMAT1 - “1,” EMAT2 - “2,” EMAT3 - “3,” EMAT4 - “4”)

Genes most differentially expressed in each cluster (Bonferroni adjusted *t*-test *p* < 0.01) included both EMT and MAT genes for all clusters (Supplementary Table S3). Similarly, the genes most associated with survival (univariable Cox regression, *p* < 0.01) included six EMT and seven MAT genes (out of 13, Supplementary Table S4). These results implicate the concurrent necessity of both signatures for cluster identification. Patient stratification using the EMT signature alone [9, 10], while successful in discerning between epithelial (CDH1, *p* = 1.4E−2) and mesenchymal (VIM, *p* = 1.8E−69) marker expressions, failed to show significantly different DSS (*p* = 0.28, Supplementary Fig. S1A). Similarly, patient stratification using the MAT gene signature alone, while successful in discerning between mesenchymal (VIM, *p* = 2.7E−7) and amoeboid marker (RHOA, *p* = 6.4E−4) expressions, failed to demonstrate significantly different DSS (*p* = 0.56, Supplementary Figure S1B).

Most breast cancer multi-gene prognostic assays (Oncotype Dx, Mammaprint, Prosigna/PAM50, Endopredict, Genomic Grade Index) prominently track proliferation biology, and it is believed that the group of proliferation-associated genes has the biggest impact on the measurement of prognosis [21]. We therefore wanted to determine whether the prognostic significance of the EMAT signature had any contribution from it possibly also tracking proliferation processes in cancer. For this purpose, we obtained a cancer proliferation gene signature consisting of 200 genes [22]. Comparing this signature with the 388-member EMAT gene signature showed a small overlap consisting of only three genes (CDH1, ELMO3, RPS6KA1). Also, the univariate Cox regression analysis did not show a significant association between these three genes and survival outcome (Supplementary Table S4). These results suggest that the prognostic impact of EMAT signature is not due to an association with proliferation and that this signature captures aggressive metastasis biology independent of proliferation biology.

### EMAT clusters provide prognostic information beyond clinical parameters or PAM50 intrinsic subtypes

Next, we examined whether the EMAT clusters are associated with clinical parameters or previously described breast cancer subtypes (Fig. 1b). Of note is the visually apparent enrichment of EMAT4, the cluster with worst survival, with triple-negative (negative for ER, PR, HER2) and basal-like (PAM50 subtype, purple) patients. In addition, most HER2-positive patients (green) appear in EMAT2. Human embryonic stem cells (hESC) have been previously shown to be associated with elevated metastasis risk [23]. Figure 1d shows that EMAT clusters display worsening prognosis proportionate to their degree of similarity to hESC, consistent with this theory. This was despite the fact that derivation of the EMAT gene signature was not designed to intentionally enrich for stem cell traits. This provides further evidence in support of the EMAT clusters representing a progressive transition from less stem-like to more stem-like cell states and less invasive to more invasive modes of cancer.

**Fig. 2:**
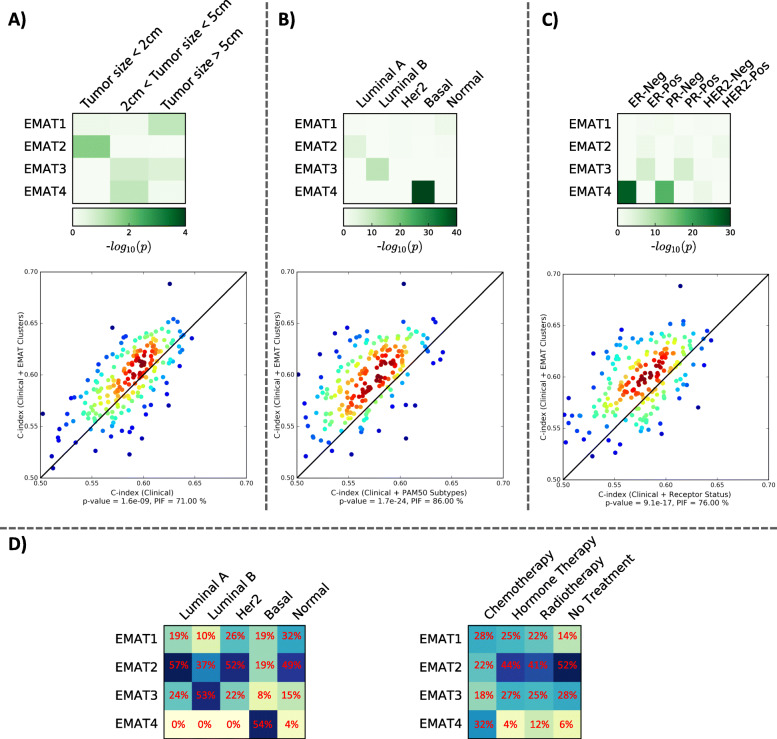
Enrichment of EMAT clusters in other breast cancer subtypes and systematic comparison of their prognostic power using cross-validation. The heatmaps show the − log10 (*p*-value) of enrichment of EMAT clusters in different subtypes or clinical parameters (using a hypergeometric test). The scatter plots compare the performance (measured in C-index) of Cox regression predictions using EMAT cluster status and clinical parameters (*y*-axis) versus other predictors (*x*-axis) (see the “Methods” section for details). If there are more points above the diagonal line (and further away from it), it shows that the method represented on the *y*-axis outperforms the method represented on the *x*-axis. The *p*-values were calculated using a one-sided Wilcoxon signed rank test and represent the significance of the improvement obtained using EMAT cluster status and clinical parameters as compared to other predictors. The measure PIF shows the percent of times in which EMAT + clinical parameters provided a more accurate prediction compared to the baseline. **a** The heatmap shows enrichment of EMAT clusters by samples of different tumor sizes. The scatter plot shows performance of Cox regression predictions using EMAT + clinical parameters versus clinical parameters alone. **b** The heatmap shows enrichment of EMAT clusters by samples of different PAM50 molecular subtypes. The scatter plot shows performance of Cox regression predictions using EMAT + clinical parameters versus PAM50 subtypes + clinical parameters. **c** The heatmap shows enrichment of EMAT clusters by samples of different receptor status. The scatter plot shows performance of Cox regression predictions using EMAT + clinical parameters versus receptor status + clinical parameters. **d** The heatmaps show the distribution of patients in each PAM50 subtypes as well as different treatments in EMAT clusters

Quantitative significance of observed associations (Supplementary Table S5) between EMAT clusters and tumor size, PAM50 subtypes, and receptor status was assessed using enrichment *p*-value (hypergeometric test, Fig. 2a–c). While the presence of small tumors in clusters with good prognosis is expected, the enrichment of EMAT1 (the cluster with the best prognosis) in large tumors suggests that large tumors do not necessarily result in poor survival in the absence of necessary metastatic mechanisms [24]. EMAT3 was enriched in ER-positive (*p* = 1.76E−06) and PR-positive (*p* = 2E−06) samples, while EMAT4 was enriched in ER-negative (*p* = 2.64E−28), PR-negative (*p* = 4.18E−18), and HER2-negative (*p* = 4.8E−3) samples. EMAT subtypes displayed enrichments for PAM50 intrinsic subtypes (“normal-like” in EMAT1, *p* = 5.59E−3, luminal A in EMAT2, *p* = 1.84E−06, luminal B in EMAT3, *p* = 7.34E−12, and basal-like in EMAT4, *p* = 7.73E−41). Despite these enrichments, however, 68% of “normal-like” samples are in clusters other than EMAT1, 43% of luminal A patients are in clusters other than EMAT2, 47% of luminal B patients are in clusters other than EMAT3, and 46% of basal patients are in clusters other than EMAT4 (Fig. 2d).

We performed Kaplan-Meier analysis of EMAT clusters within each PAM50 subtype (Supplementary Figure S2) and within HER2+ and triple negative (TN) subtypes (Supplementary Figure S3). The results revealed that survival outcomes of EMAT subtypes within each of these subtypes are generally consistent with their survival outcomes when all samples are analyzed simultaneously, with EMAT4 having poor prognosis and EMAT1 having a better prognosis. A similar observation was made when we evaluated survival behavior of EMAT clusters in treatment naïve and treated patients (Supplementary Figure S4). It is worth mentioning that the survival of treatment naïve versus treated patients did not show a statistically significant difference within each EMAT cluster, even though the treated patients showed a better 10-year survival probability (Supplementary Figure S5).

To ascertain whether EMAT clustering provides prognostic information beyond what is captured by the intrinsic subtypes, we trained a Cox regression model (CRM) on the LNN samples from the METABRIC dataset using four types of predictors: (1) clinical parameters (CP, including age, tumor size, tumor grade, adjuvant chemotherapy, hormone therapy, or radiotherapy), (2) CP and receptor status, (3) CP and PAM50 subtype status, and (4) CP and EMAT cluster status. The Cox regression model trained on CP and EMAT cluster status had the smallest *p*-value using a likelihood ratio test (*p* = 6.6E−5 compared to *p* = 1.0E−2 for CP and receptor status, *p* = 1.5E−2 for CP and PAM50 subtypes, and *p* = 4.2E−3 for CP only). However, because EMAT cluster status was defined based on samples used in the CRMs, while the other subtypes were defined a priori, this result while promising was not conclusive.

To rigorously compare the predictive ability of the various groups while removing the effect of the varying number of predictive features, and also to test the generalizability of these models on unseen data, we next used a cross-validation framework (see Methods for details). Samples were randomly divided in two groups of (almost) equal size and a CRM trained on one half was used to estimate the expected survival on the other half. This process was repeated 200 times, each time using a distinct random partition of data. The CP and EMAT cluster status CRM provided the best predictions (Fig. 2a–c bottom panels), evaluated using a one-sided Wilcoxon signed rank test on paired C-index values of the compared methods as well as another measure called percentage of improved folds (PIF) [25] defined as percent of the partitions in which one class of features outperforms another class. Thus, although some of the EMAT clusters are enriched in previously known molecular subtypes of breast cancer (e.g., PAM50), they are quite distinct from such subtypes (Fig. 2d). In addition, the EMAT clusters are better predictors of patient survival outcome than PAM50 or receptor-based subtypes. Finally, even though CP including the type of treatment are important in predicting survival outcome, the EMAT clusters are not simply surrogate measures of adjuvant treatments (Fig. 2d) but rather provide extra information that are useful in predicting patient prognosis, as is evident from Fig. 2a.

### Cross-dataset validation of the prognostic value of EMAT cluster designation

**Fig. 3:**
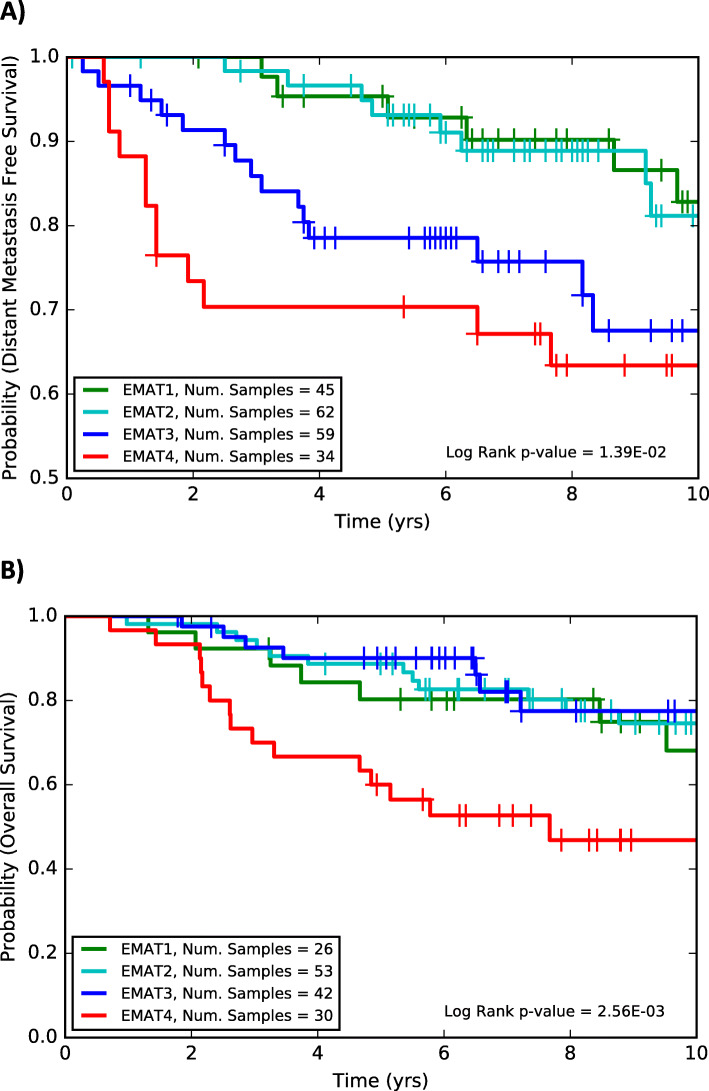
The Kaplan-Meier survival plots and biomarker status for EMAT subtypes of LNN breast cancer samples from the GSE11121 (**a**) and NKI295 (**b**) datasets using cross-dataset analysis. A centroid-based classifier trained on LNN METABRIC samples is used to assign EMAT subtype labels to each sample

We next evaluated the prognostic power of the EMAT clusters on independent, lymph node negative datasets (151 LNN patients in the NKI295 dataset and GSE11121), one of which was a treatment-naive dataset [26] (GEO accession number: GSE11121), and stratified patients according to their EMAT subtypes using a centroid-based classifier, which was trained using LNN METABRIC samples (see the “Methods” section). The GSE11121 dataset contains distant metastasis-free survival (DMFS) information, allowing us to specifically study the ability of EMAT clusters to predict metastasis occurrence. EMAT subtype Kaplan-Meier curve separation was statistically significant (*p* = 2.56E−03, log rank test, 151 LNN cohort in NKI 295, and *p* = 1.39E−2, log rank test, GSE11121, Fig. 3). In addition, univariable and multivariable Cox regression analysis (when considering CP) showed that these clusters provide a statistically significant prognostic value in these independent datasets as well which comprised only of LNN patients where none of the patients received any adjuvant chemotherapy at all (GSE11121, Supplementary Table S6) or only a small fraction (6 patients) received adjuvant chemotherapy (151 LNN cohort in NKI295, Supplementary Table S6). Survival behavior of EMAT subtypes remains largely similar to their behavior in the LNN METABRIC dataset, with EMAT4 having the worst survival, EMAT3 the second worst survival with EMAT1 and EMAT2 the best survival probabilities (see also Supplementary Figure S6). These results show a high concordance between the characteristics of the EMAT subtypes in three independent datasets.

### Identification of transcription factors associated with EMAT clusters

**Fig. 4:**
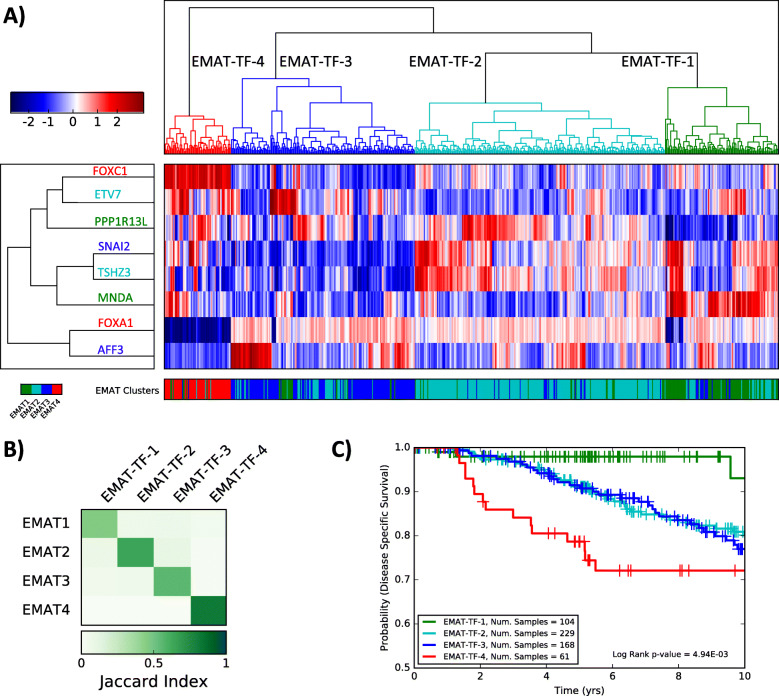
Analysis of clusters obtained using eight TFs most under-expressed or over-expressed in each EMAT cluster. **a** Hierarchical clustering based on the expression of the eight identified TFs is used to cluster samples into four groups. The color bar at the bottom shows the true EMAT cluster label of each sample. **b** Concordance of clusters obtained using eight TFs with EMAT clusters based on the Jaccard index. **c** Kaplan-Meier survival plots for clusters obtained using the TFs

We evaluated the expression of 1338 transcription factors (TFs) present in the METABRIC dataset (Supplementary Table S7) and utilized a *t*-test (corrected for multiple hypothesis testing) to identify TFs that are differentially expressed in one cluster compared to others, in LNN samples. We identified PPP1R13L and MNDA (EMAT1), ETV7 and TSHZ3 (EMAT2), SLUG and AFF3 (EMAT3), and FOXA1 and FOXC1 (EMAT4), which were under-expressed and over-expressed in each cluster, respectively. Hierarchical clustering of LNN samples from the METABRIC dataset using the eight identified TFs had a very high concordance with clusters obtained using the full EMAT gene signature. In addition, Kaplan-Meier analysis using the clusters defined using these TFs demonstrated a significant and similar separation of survival curves (Fig. 4), lending support to their potential clinical utility as biomarkers of the identified EMAT clusters.

## Discussion and conclusion

The clinical significance and necessity of EMT in driving metastasis has been questioned in recent publications [27–30]. While the findings in such studies have been challenged to be inconclusive [31, 32], there is another possible explanation for the reported findings without upending the potential significance of EMT in metastasis. When a cancer cell proceeds along EMT, microenvironmental pressures (e.g., hypoxia) or xenobiotic exposure (e.g., chemotherapy or EMT-targeted biologic therapy) may lead to an increase in the entropy of the EMT path of cellular transformation. A cancer cell of epithelial origin that has already undergone EMT when faced with such a situation may then undergo plastic transformation by a process such as MAT and adopt a low entropy, alternate amoeboid motility program to metastasize [12]. Indeed simultaneous targeting of both mesenchymal and amoeboid motility in an animal model of cancer progression has been demonstrated to effectively arrest metastatic spread [13]. Only when both processes are considered together does the prognostic value of such distinct phenotypes become demonstrable.

As LNN breast cancer patients are typically perceived to harbor a lower metastasis risk compared to lymph node-positive breast cancer patients, many LNN patients (especially those diagnosed with ER+ breast cancer) are not recommended to be treated with adjuvant chemotherapy. We therefore used lymph node negativity as a criterion to select clinical samples for analysis to ensure that the samples obtained were from early clinical stages of the metastasis cascade. Furthermore, this would help fulfill the greater unmet clinical need of identifying atypical LNN breast cancer patients who possibly harbor elevated metastasis risk, as indicated by an EMAT3 or EMAT4 cluster designation, and may potentially benefit from life-extending adjuvant chemotherapy. Similarly, patients diagnosed with LNN HER2+ breast cancer or LNN TN breast cancer are known to harbor greater metastatic recurrence risk than patients diagnosed with LNN ER+ breast cancer, for which reason many such patients are typically recommended to be treated with adjuvant chemotherapy. In this situation identifying atypical LNN HER2+ or TN breast cancer patients who possibly harbor lower metastatic recurrence risk, as indicated by an EMAT1 cluster designation, would also be important as such patients may not require treatment with adjuvant chemotherapy and thereby avoid unnecessary chemotherapeutic side effects.

Limitations of the present study include our inability to consider the potential impact of the host immune response, tumor stromal factors, and/or that of non-cancer cells of the tumor microenvironment on influencing metastatic proclivity and prognostic prediction thereof. Because of the retrospective design of the study, reported results while suggestive are not conclusive regarding the potential clinical impact of the EMAT signature. As the dataset patients were treated at different time-points, conclusions cannot be generalized because the distribution of clinical characteristics may be different in patients from other areas (even within the same geographical region). The current study therefore suffers from biases inherent to such a study design. Our results do however suggest that they merit further investigation. To this end, the centroid-based EMAT classifier developed in the present study can be used as a single sample predictor to determine the EMAT cluster designation status of new patient samples, even if they were not included in the present study.

Our study revealed the existence of breast cancer progression and metastasis subtypes that display a dynamic overlap of EMT and MAT rather than discrete E-, M-, and A-like clusters, emphasizing the advantage of using the EMAT signature over using only E, M, or A biomarkers, to distinguish patient groups with distinct prognosis. What is important to note is that the metastatic propensity of breast cancer cannot be accurately captured or predicted by consideration of clinical stage, receptor status, PAM50 molecular subtype status, or treatment variables alone and that the additional consideration of metastasis biology-based predictors is warranted.

## Supplementary information

**Additional file 1: Figure S1.** Kaplan-Meier survival analysis corresponding to clusters of LNN METABRIC samples based on EMT and MAT signatures. (A) Kaplan-Meier survival analysis for clusters obtained based on EMT gene signature using hierarchical clustering. (B) Kaplan-Meier survival analysis for clusters obtained based on MAT gene signature using hierarchical clustering. **Figure S2.** Kaplan-Meier survival analysis of EMAT clusters within each PAM50 subtypes of LNN METABRIC samples. **Figure S3.** Kaplan-Meier survival analysis of EMAT clusters within HER2-positive and triple negative (TN) subtypes of LNN METABRIC samples. **Figure S4.** Kaplan-Meier survival analysis of EMAT clusters within treatment-naïve and treated patients of LNN METABRIC samples. **Figure S5.** Kaplan-Meier survival analysis of treatment-naïve versus treated patients of LNN METABRIC samples within each EMAT cluster. **Figure S6.** Cross-dataset analysis. The Kaplan-Meier survival plots correspond to EMAT subtypes of LNN breast cancer samples from the GSE11121 dataset. A 5-NN classifier trained on LNN METABRIC samples is used to assign EMAT subtype labels to each sample. In the figure, C1 = EMAT1, C2 = EMAT2, C3 = EMAT3 and C4 = EMAT4.

**Additional file 2: Table S1.** List of genes in the EMAT, EMT and MAT signatures. The table content is provided as a separate xlsx file.

**Additional file 3: Table S2.** EMAT cluster labels of samples in the METABRIC, GSE11121 and NKI295 datasets. The table content is provided as a separate xlsx file. The labels are obtained using hierarchical clustering with 4 clusters, as described in the manuscript.

**Additional file 4: Table S3.** Percent of EMT and MAT genes present among differentially expressed genes (DEGs) for each cluster. The table content is provided as a separate xlsx file. DEGS for each EMAT cluster were defined as differentially expressed in that cluster compared to other clusters (Bonferroni adjusted *p* < 0.01, using a two-sided t-test).

**Additional file 5: Table S4.** The association of EMAT genes with survival outcome. The *p*-values are obtained using a univariable Cox regression analysis.

**Additional file 6: Table S5.** A summary of the characteristics of the EMAT clusters obtained using lymph node-negative breast cancer patients from the METABRIC study. In this table, P stands for positive and N for negative. EMAT1 has the least similarity to hESC and is enriched in normal-like PAM50 subtype of breast cancer and has a good prognosis. EMAT2, the cluster with a relatively good prognosis, has little similarity to hESC, is enriched in Luminal A subtype and in ER-positive and PR-positive samples. EMAT3, the cluster with a relatively moderate prognosis, has a high degree of similarity to hESC, is enriched in Luminal B subtype and in ER-positive, PR-positive and HER2-negative samples. EMAT4, the cluster with the worst prognosis, shows the highest degree of similarity to hESC, is enriched in the basal-like subtype of breast cancer as well as ER-negative, PR-negative and HER2-negative samples.

**Additional file 7: Table S6.** Univariable and multivariable Cox regression analysis for validation dataset samples from GSE11121 and NKI295. The table content is provided as a separate xlsx file.

**Additional file 8: Table S7** Differential expression analysis of TFs for each EMAT cluster. The table content is provided as a separate xlsx file. The *p*-values were obtained using a two-sided t-test and were corrected for multiple hypothesis testing.

## Data Availability

Gene expression and clinical data for 562 LNN breast cancer patient samples from the METABRIC study is available from (OASIS http://oasis-genomics.org/, [15]) and for two independent datasets of 200 LNN samples and 151 LNN samples are available at (http://www.ncbi.nlm.nih.gov/geo/ GEO accession number GSE11121, http://ccb.nki.nl/data/ [16]), as summarized in Table 1. Gene expression profiles of H1 hESC lines [17] are available at (GEO accession number GSE54186).

## Abbreviations

A: Amoeboid
C-index: Concordance index
CP: Clinical parameters
CRM: Cox regression model
DMFS: Distant metastasis-free survival
DSS: Disease-specific survival
E: Epithelial
EMAT: Epithelial-to-mesenchymal-to-amoeboid transition
EMT: Epithelial-to-mesenchymal transition
hESC: Human embryonic stem cells
KNN: K-nearest neighbor
LNN: Lymph node-negative
LNNBC: Lymph node-negative breast cancer
LP: Lymph node-positive
M: Mesenchymal
MAT: Mesenchymal-to-amoeboid transition
PAM50: Prediction Analysis of Microarray 50
PIF: Percentage of improved folds (PIF)
TF: Transcription factor
