# Supplementary material for: Superior breast cancer metastasis risk stratification using an epithelial-mesenchymal-amoeboid transition gene signature
Source: Breast Cancer Res. 2020 Jul 8;22:74. doi: 10.1186/s13058-020-01304-8 (PMC7341640; doi:10.1186/s13058-020-01304-8)
Supplement: Supplementary file 1 — Additional file 1: Figure S1. Kaplan-Meier survival analysis corresponding to clusters of LNN METABRIC samples based on EMT and MAT signatures. (A) Kaplan-Meier survival analysis for clusters obtained based on EMT gene signature using hierarchical clustering. (B) Kaplan-Meier survival analysis for clusters obtained based on MAT gene signature using hierarchical clustering. Figure S2. Kaplan-Meier survival analysis of EMAT clusters within each PAM50 subtypes of LNN METABRIC samples. Figure S3. Kaplan-Meier survival analysis of EMAT clusters within HER2-positive and triple negative (TN) subtypes of LNN METABRIC samples. Figure S4. Kaplan-Meier survival analysis of EMAT clusters within treatment-naïve and treated patients of LNN METABRIC samples. Figure S5. Kaplan-Meier survival analysis of treatment-naïve versus treated patients of LNN METABRIC samples within each EMAT cluster. Figure S6. Cross-dataset analysis. The Kaplan-Meier survival plots correspond to EMAT subtypes of LNN breast cancer samples from the GSE11121 dataset. A 5-NN classifier trained on LNN METABRIC samples is used to assign EMAT subtype labels to each sample. In the figure, C1 = EMAT1, C2 = EMAT2, C3 = EMAT3 and C4 = EMAT4. [file 13058_2020_1304_MOESM1_ESM.pdf]

Supplementary Material for "Superior breast cancer metastasis risk stratification using an Epithelial-mesenchymal-amoeboid transition gene signature"

Amin Emad[1,2,3], Tania Ray[4], Tor W. Jensen[5,7], Meera Parat[3], Rachael Natrajan[6], Saurabh Sinha[2,3,7], Partha S. Ray[4]

[1] Department of Electrical and Computer Engineering, McGill University, Canada

[2] Carl R. Woese Institute for Genomic Biology, University of Illinois at Urbana-Champaign, USA

[3] Department of Computer Science, University of Illinois at Urbana-Champaign, USA

[4] Onconostic Technologies, Inc., Champaign, Illinois, USA

[5] Illinois Health Sciences Institute, University of Illinois at Urbana-Champaign, USA

[6] The Breast Cancer Now Toby Robins Research Centre, The Institute of Cancer Research, London, UK

[7] Cancer Center at Illinois, University of Illinois at Urbana-Champaign, USA

Corresponding Authors:

Partha S. Ray
Onconostic Technologies, Inc.
60 Hazelwood Drive, Suite 208
Champaign, IL, USA 61820
Phone: (+1) 908-625-5169
Email: partha.ray@onconostictechnologies.com

Saurabh Sinha
2122 Siebel Center
201 N. Goodwin Ave
Urbana, IL, USA 61801
Phone: (+1) 217-333-3233
Email: sinhas@illinois.edu

Supplementary Figures:

A) B)

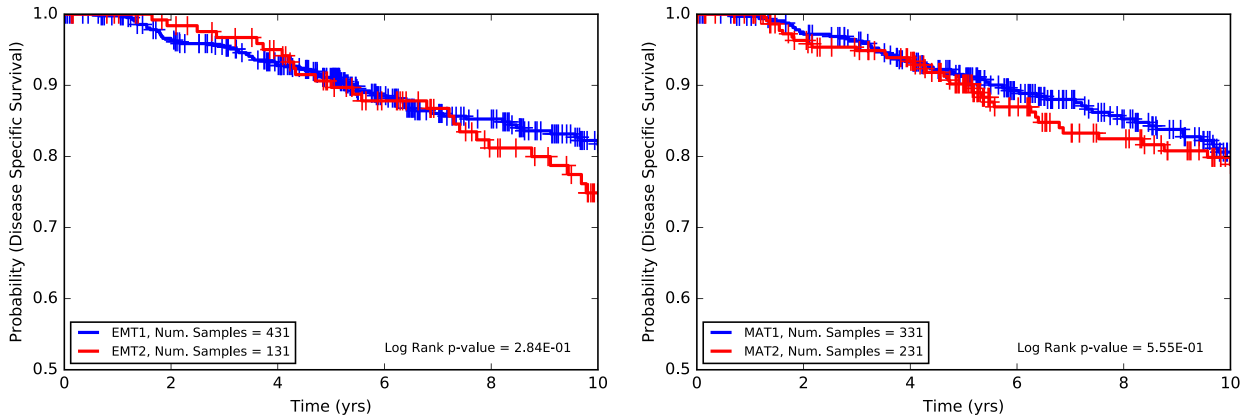

Figure S1: Kaplan-Meier survival analysis corresponding to clusters of LNN METABRIC samples based on EMT and MAT signatures. (A) Kaplan-Meier survival analysis for clusters obtained based on EMT gene signature using hierarchical clustering. (B) Kaplan-Meier survival analysis for clusters obtained based on MAT gene signature using hierarchical clustering.

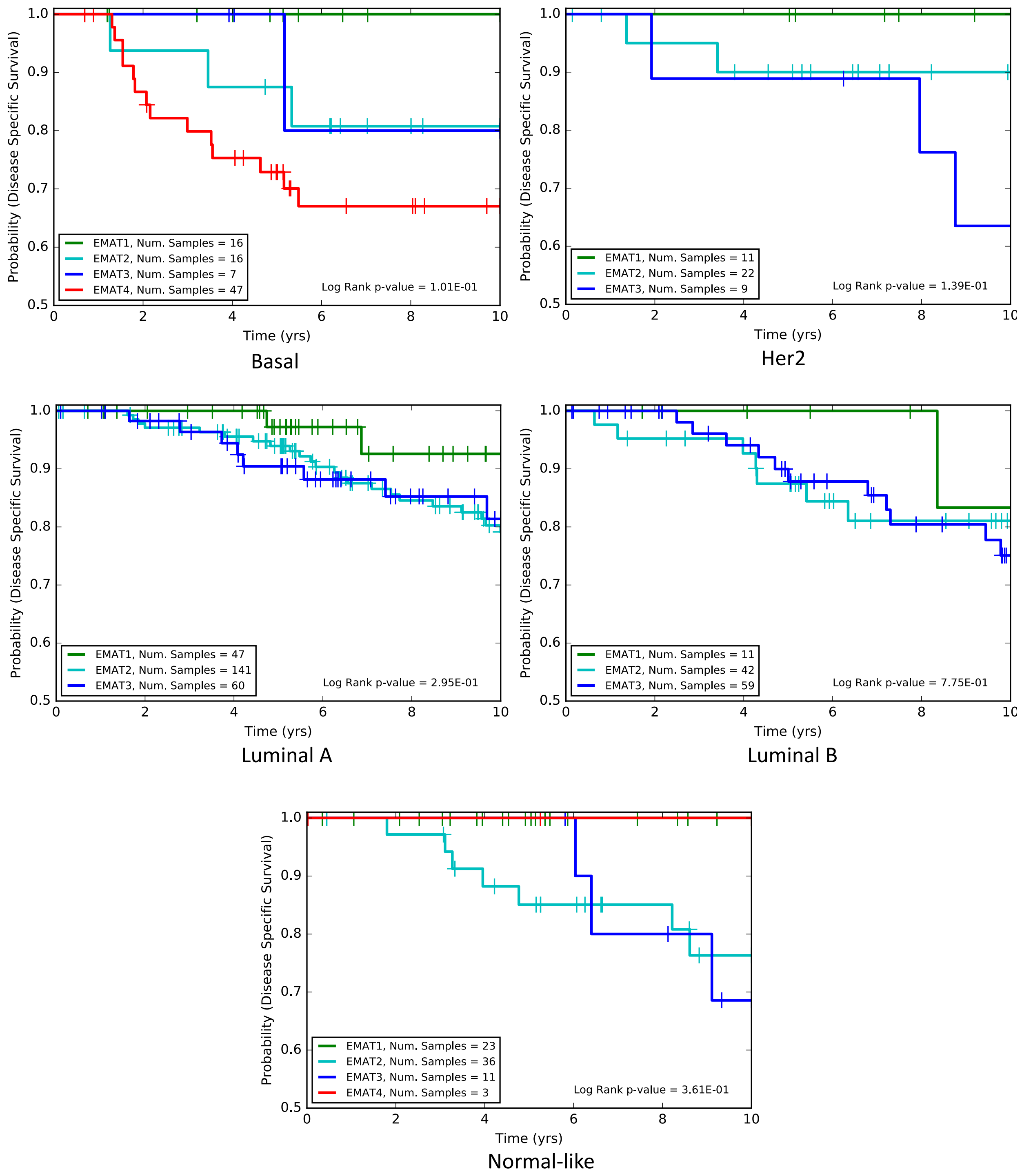

Figure S2: Kaplan-Meier survival analysis of EMAT clusters within each PAM50 subtypes of LNN METABRIC samples.

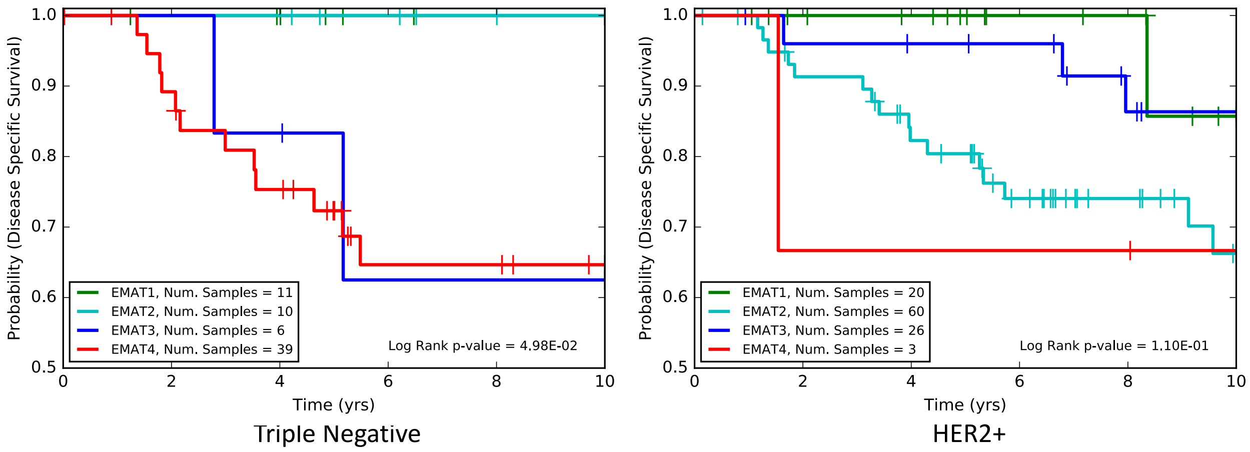

Figure S3: Kaplan-Meier survival analysis of EMAT clusters within HER2-positive and triple negative (TN) subtypes of LNN METABRIC samples.

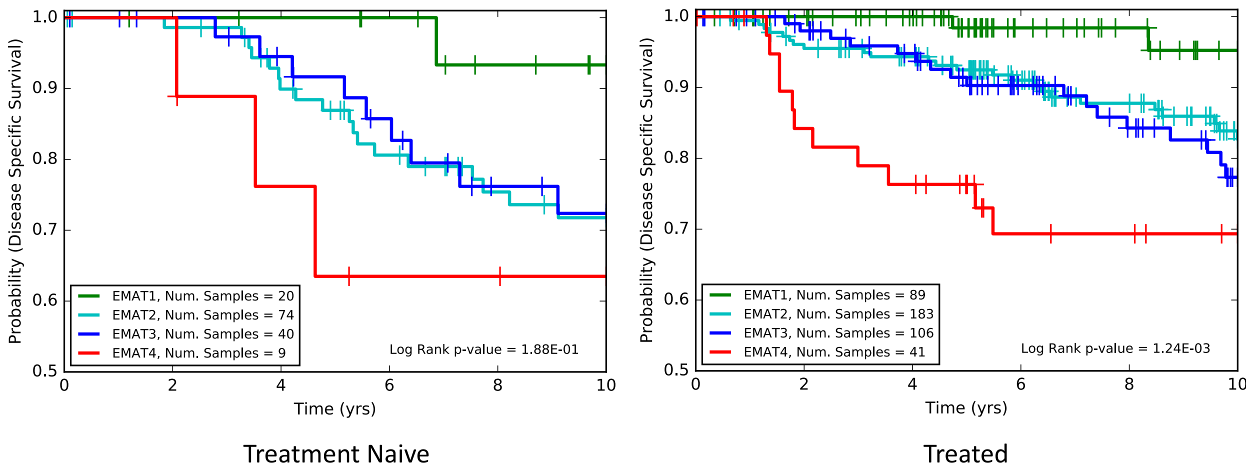

Figure S4: Kaplan-Meier survival analysis of EMAT clusters within treatment-naïve and treated patients of LNN METABRIC samples.

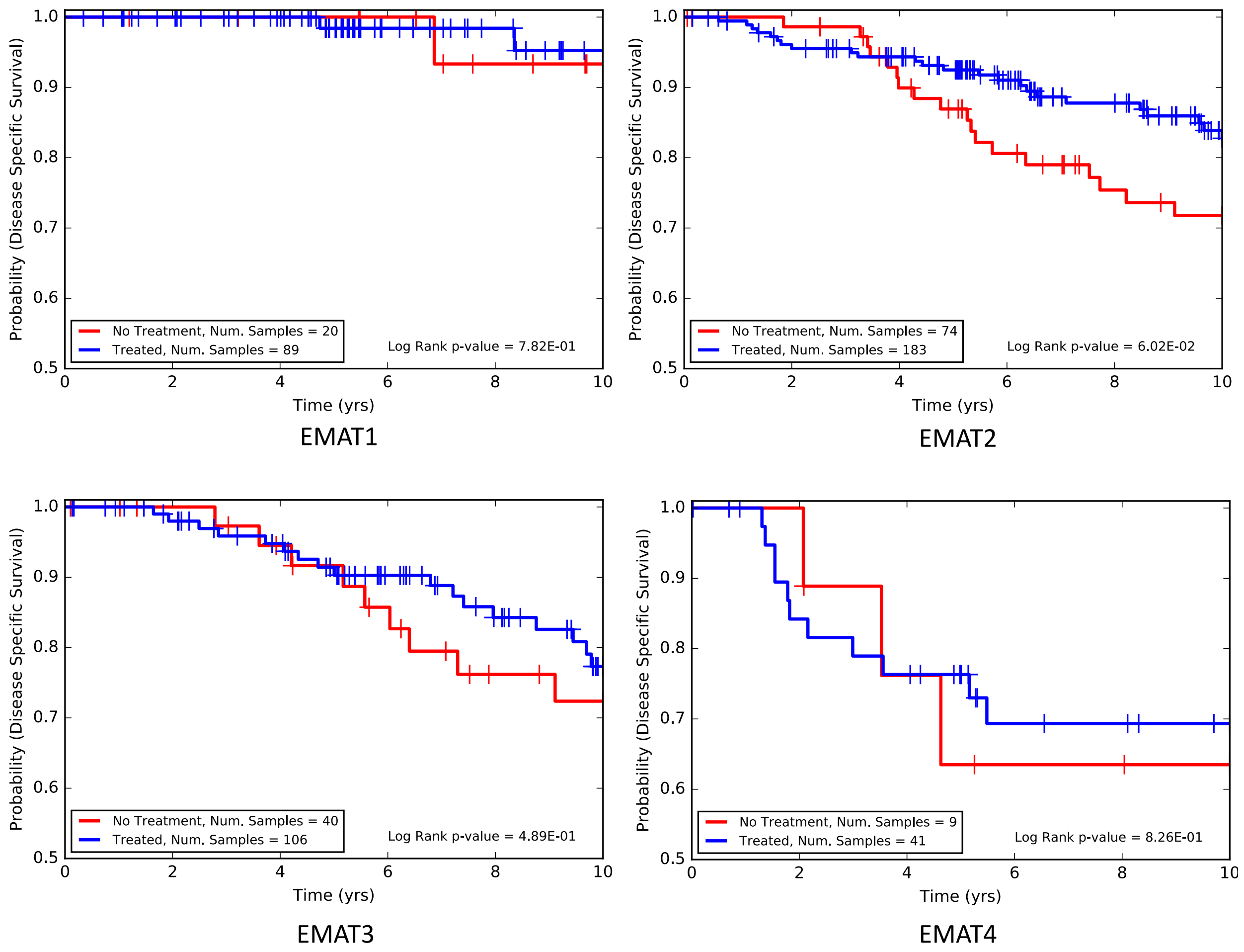

Figure S5: Kaplan-Meier survival analysis of treatment-naïve versus treated patients of LNN METABRIC samples within each EMAT cluster.

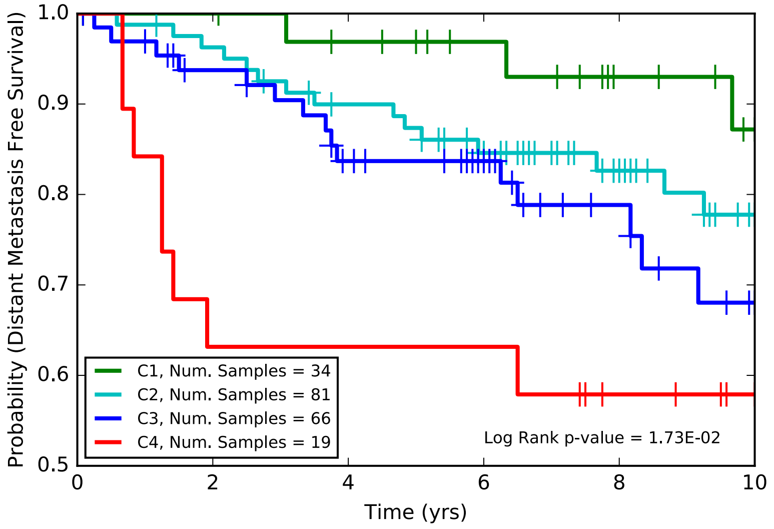

Figure S6: Cross-dataset analysis. The Kaplan-Meier survival plots correspond to EMAT subtypes of LNN breast cancer samples from the GSE11121 dataset. A 5-NN classifier trained on LNN METABRIC samples is used to assign EMAT subtype labels to each sample. In the figure, C1 = EMAT1, C2 = EMAT2, C3 = EMAT3 and C4 = EMAT4.

Legends of Supplementary Tables:

Table S1: List of genes in the EMAT, EMT and MAT signatures. The table content is provided as a separate xlsx file.

Table S2: EMAT cluster labels of samples in the METABRIC and GSE11121 datasets. The table content is provided as a separate xlsx file. The labels are obtained using hierarchical clustering with 4 clusters, as described in the manuscript.

Table S3: Percent of EMT and MAT genes present among differentially expressed genes (DEGs) for each cluster and the ranked list of EMAT genes based on their differential expression p-values. The table content is provided as a separate xlsx file. DEGS for each EMAT cluster were defined as differentially expressed in that cluster compared to other clusters (Bonferroni adjusted $p<0.01$ using a two-sided t-test in the first tab).

Table S4: The association of EMAT genes with survival outcome. The p-values are obtained using a univariable Cox regression analysis.

Table S5: A summary of the characteristics of the EMAT clusters obtained using lymph node-negative breast cancer patients from the METABRIC study. In this table, P stands for positive and N for negative. EMAT1 has the least similarity to hESC and is enriched in normal-like PAM50 subtype of breast cancer and has a good prognosis. EMAT2, the cluster with a relatively good prognosis, has little similarity to hESC, is enriched in Luminal A subtype and in ER-positive and PR-positive samples. EMAT3, the cluster with a relatively moderate prognosis, has a high degree of similarity to hESC, is enriched in Luminal B subtype and in ER-positive, PR-positive and HER2-negative samples. EMAT4, the cluster with the worst prognosis, shows the highest degree of similarity to hESC, is enriched in the basal-like subtype of breast cancer as well as ER-negative, PR-negative and HER2-negative samples.

Table S6: Univariable and multivariable Cox regression analysis for GSE11121 samples. The table content is provided as a separate xlsx file.

Table S7: Differential expression analysis of TFs for each EMAT cluster. The table content is provided as a separate xlsx file. The p-values were obtained using a two-sided t-test and were corrected for multiple hypothesis testing.
